# Exploring the Mechanism of Ling-Gui-Zhu-Gan Decoction in Ventricular Remodeling after Acute Myocardial Infarction Based on UPLC and *In Vivo* Experiments

**DOI:** 10.1155/2022/8593176

**Published:** 2022-05-16

**Authors:** Peng Zhou, Meng Zhang, Xiao-ni Zhao, Tong-juan Tang, Xiang Wang, Lu-lu Huang, Qi Kong, Liang Wang, Jin-ling Huang

**Affiliations:** ^1^School of Integrated Chinese and Western Medicine, Anhui University of Chinese Medicine, Hefei 230012, China; ^2^Institute of Integrated Chinese and Western Medicine, Anhui Academy of Chinese Medicine, Hefei 230012, China; ^3^Anhui Province Key Laboratory of Chinese Medicinal Formula, Hefei 230012, China

## Abstract

Ventricular remodeling (VR) after acute myocardial infarction (AMI) is an important pathophysiological basis for the development of chronic heart failure (CHF). At present, Ling-Gui-Zhu-Gan decoction (LGZGD) has been widely reported in the clinical treatment and basic research of cardiovascular diseases (CVDs), such as myocardial infarction, heart failure, and angina pectoris. However, the mechanism of LGZGD against VR after AMI remains unclear. Ultra-performance liquid chromatography (UPLC) was applied to investigate the major constituents of LGZGD, and molecular docking was used to predict the targets on the NLRP3/Caspase-1/GSDMD signaling pathway. *In vivo*, histological changes in the myocardium were visualized using HE staining and Masson staining, and cardiomyocyte apoptosis was detected using TUNEL. IL-1*β* activity in rat serum was determined by ELISA. Finally, NLRP3, Caspase-1, and GSDMD expressions were analyzed through RT-qPCR and Western blotting. The results showed that 8 authentic reference substances have been detected in LGZGD. Molecular docking showed that the major chemical constituents of LGZGD had a good binding activity with NLRP3, Caspase-1, and GSDMD. Our results showed that LGZGD treatment markedly improved cardiac pathology, decreased cardiomyocyte apoptosis, reduced IL-1*β* activity, and regulated the expression of genes and proteins related to the NLRP3/Caspase-1/GSDMD signal pathway. These results suggest that LGZGD protects against VR after AMI through NLRP3/Caspase-1/GSDMD signal pathway.

## 1. Introduction

Acute myocardial infarction (AMI) is a prominent illness with a high morbidity and mortality rate globally, which is defined by long-term ischemia and death following coronary artery obstruction and harms patients' health [1]. Ventricular remodeling (VR) following AMI is a significant pathophysiological factor in the development of chronic heart failure (CHF) [2]. Restoring myocardial blood flow in a timely and effective manner is the basis of AMI therapy [3]. However, reperfusion therapy cannot be treated in time and effectively and can also cause myocardial injury, ischemia/reperfusion injury [4]. Therefore, seeking active drug treatment, especially the multi-target treatment of traditional Chinese medicine compounds, can significantly reduce AMI.

The activation of the nucleotide-binding domain and leucine-rich repeat protein 3 (NLRP3) inflammasomes might result in the production of proinflammatory cytokines such as interleukin-1*β* (IL-1*β*). NLRP3 overexpression is a major cause of coronary artery disease (CAD) and myocardial ischemia-reperfusion (I/R) damage [5]. Numerous basic and clinical studies have shown that inhibiting the activation of the NLRP3 inflammasome in the early stage of reperfusion after AMI can reduce the overall size of the infarction and maintain normal cardiac function [6, 7]. Experimental studies have shown that NLRP3 inflammasome targeted therapy may be a feasible strategy to reduce infarct size and prevent heart failure after AMI [8]. The present study shows oxidative stress injury, and inflammatory response can lead to the activation of the NLRP3 inflammasome, resulting in a cascade of inflammatory responses. Therefore, inhibition of oxidative stress injury or inflammatory response can inhibit the formation of NLRP3 inflammasome [9, 10]. However, no selective NLRP3 inhibitors are clinically available at present. Therefore, it is important to find safe and effective NLRP3 inflammasome inhibitors from Chinese Medicine, to improve myocardial ischemia resistance and reduce the loss of myocardial cells.

Ling-Gui-Zhu-Gan decoction (LGZGD), a traditional Chinese medicine, has been used to treat various diseases, including cardiovascular disease, Alzheimer's disease, and diabetes [11–14]. Previous studies found that the combination of LGZGD and metoprolol is safe and feasible [15]. LGZGD is composed of four crude drugs, namely, *Poria cocos* (Schw.) Wolf, *Cinnamomi Ramulus*, *Rhizoma Atractylodis Macrocephalae*, and *Radix Glycyrrhizae* [16]. Modern pharmacological studies revealed that LGZGD has been widely reported in the clinical prevention and treatment of CVDs, such as arrhythmia, cardiac failure, and angina pectoris [17, 18]. Our previous study found that LGZGD has good anti-inflammatory activity, specifically manifested in improving the structure and function of the heart by regulating IL-1*β* and IL-6 in the AMI rat model [19]. A recent study has discovered that LGZGD possesses significant anti-inflammatory and antioxidant properties. *In vivo*, LGZGD has been shown to ameliorate myocardial damage and prevent VR via modulating the nuclear factor kappa-B (NF-*κ*B) signaling pathway [20]. LGZGD can effectively inhibit the overactivation of cytokines by regulating the production of proteins involved in the IKK/I-*κ*B/NF-*κ*B signaling pathway [21]. In addition, LGZGD could enhance the activity of glutathione peroxidase (GSH-Px) and superoxide dismutase (SOD) in cardiomyocytes via H_2_O_2_-induced injury, reduce the levels of malonic dialdehyde (MDA) and lactic dehydrogenase (LDH), and inhibit cardiomyocyte apoptosis, which showed that the antioxidant mechanism was related to the Nrf2/Keap1/HO-1 pathway [22, 23]. However, whether the active compounds of LGZGD act on the NF-*κ*B downstream target (NLRP3) and which can be the lead compounds are not well understood. Therefore, molecular docking was applied to predict the mechanism of action and search for lead compounds of NLRP3, and then its action mechanism was further verified by animal experiments *in vivo*.

Based on the antioxidant and anti-inflammatory effects of LGZGD in our previous studies, we will further study its effect on the downstream NLRP3 signaling pathway. In this study, the main chemical constituents of LGZGD were qualitatively identified via UPLC, and molecular docking was used to predict the relationship between the major chemical constituents of LGZGD and the relevant proteins of NLRP3/Caspase-1/GSDMD signaling pathway and explore molecular mechanism of LGZGD with a focus on the NLRP3/Caspase-1/GSDMD signaling pathway in AMI-induced rat model.

## 2. Materials and Methods

### 2.1. Chemicals and Materials

Liquiritin, isoliquiritin, cinnamic acid, glycyrrhizic acid, coumarin, cinnamic alcohol, cinnamaldehyde, and atractylenolide III were obtained from Shanghai Yuanye Bio-Technology Company (Shanghai, China). The purity of all standard compounds has met the analytical requirements (>98%). The four herbs of LGZGD were purchased from Anhui Puren Chinese Medicine Co., Ltd. (Bozhou China). Acetonitrile and methanol were of chromatographic grades (Merck Company, Germany). Rat IL-1*β* ELISA kit was purchased from Meimian (Jiangsu, China). Antibodies (NLRP3, GSDMD, GAPDH) were purchased from Abcam (Cambridge, MA, USA). Caspase-1 p20 was purchased from Affinity Biosciences (Cincinnati, OH, USA).

### 2.2. Extraction of LGZGD

Powdered LGZGD was prepared according to the following methods. Briefly, crude materials of *Cinnamomi Ramulus* (9 g) and *Rhizoma Atractylodis Macrocephalae* (9 g) were boiled, and the essential oil and aromatic water were collected; then, the mixture of gruff materials and two other crude materials of *Poria cocos* (Schw.) Wolf (12 g) and *Radix Glycyrrhizae* (6 g) were immersed in water for 30 min and boiled for 1.5 h three times with 8-fold water. After filtration, the filtrate was combined three times, then concentrated, and finally evaporated to dryness to obtain LGZGD powder.

### 2.3. Analysis of LGZGD by UPLC-PDA

The main active components of LGZGD were qualitatively identified by Waters ACQUITY UPLC H-Class system (Waters, Milford, MA). ACQUITY BEH C18 column specifications were selected as 2.1 mm × 100 mm, 1.7 *μ*m (Waters, Milford, MA). The mobile phase was 0.05% (v/v) phosphoric acid-distilled water (A) and acetonitrile (B) system, followed by gradient elution: 5–33% B for 0–8 min; 33–95% B for 8–16 min; 95–5% B for 16–18 min. The setting parameters were as follows: column temperature = 30°C; flow rate = 0.2 mL/min; injection volume = 1 *μ*L; wavelength = 210 nm.

### 2.4. Molecular Docking Prediction of Major Components of LGZGD

CB-DOCK was used to conduct molecular docking between ligand and receptor. The 3D structures of NLRP3, Caspase-1, and GSDMD were downloaded from RCSB (https://www.rcsb.org/) [24], and the PDB IDs were 6npy [25], 1rwx [26], and 5wqt [27]. PDB formats of related proteins and major components of LGZGD were input to CB-Dock [28]. The lower the Vina score is, the more stably the ligand binds to the receptor. Considering that the binding sites were as consistent as possible, the docking results of NLRP3, Caspase-1, and GSDMD inhibitors MCC950, ML132, and LDC7559 were selected as positive references.

### 2.5. In Vivo Experiment

#### 2.5.1. Animals

Male Sprague-Dawley rats (weighing 200 ± 20 g) were obtained from Anhui Medical University's Experimental Animal Center (SCXK 2017–001). All experiments met the requirements pertaining to the handling of animals and thus were approved by the Experimental Animal Ethics Committee of Anhui University of Chinese Medicine.

#### 2.5.2. Establishment of AMI Rat Model

As previously mentioned, the model of AMI in rats was constructed by ligating the left anterior descending (LAD) coronary artery [29]. Briefly, all rats were anesthetized with isoflurane and equipped with respirators to reduce the pain. Under the previous condition, the left thorax of the rat was opened and ligated 2 mm below the left atrial appendage with 6–0 silk thread. Rats in the sham group underwent thoracotomy perforation without ligature.

#### 2.5.3. Drug Treatment

Two weeks later, rats with AMI were randomly assigned to four groups: model (i.e., deionized water), LGZGD (i.e., 4.2 g/kg, equivalent to the dry weight of the raw materials), and captopril (i.e., 3.375 mg/kg), for 4 weeks; rats in the sham group received the same volume of deionized water intragastrically.

#### 2.5.4. Hemodynamic Indexes' Assessment

After the last treatment, LVSP, LVEDP, +dP/dtmax, and −dP/dtmax were identified and statistically evaluated using PowerLab (Castle Hill, Australia).

#### 2.5.5. Myocardial Histopathology

The left ventricular myocardium's myocardial infarction region was collected, fixed with 4% paraformaldehyde, and paraffin-embedded tissue was processed into thick tissue slices. Hematoxylin-eosin (HE) staining and Masson staining were used to observe the pathological changes, collagen deposition, and collagen volume fraction (CVF).

#### 2.5.6. TUNEL Staining

The TdT-mediated dUTP nick-end labeling (TUNEL) technique was used to detect the free 3′-OH chain breaks caused by DNA degradation [30]. Under the microscope, five visual fields were randomly selected from each slice; in these fields, we counted the numbers of all cardiomyocytes and apoptotic cardiomyocytes. The calculation formula is as follows: apoptosis rate = the number of apoptotic cardiomyocytes/number of all cardiomyocytes × 100%.

#### 2.5.7. Immunohistochemical Analyses

Left ventricle samples were stained with primary antibodies against NLRP3 (1 : 100), Caspase-1 (1 : 100), and GSDMD (1 : 1000) and were incubated overnight at 4°C, followed by secondary antibodies (1 : 200). Next, the sections were stained with DAB. Finally, a fluorescence microscope was used to obtain images of the tissues.

#### 2.5.8. ELISA for Serum Levels of IL-1*β*

IL-1*β* level in rat serum was detected by commercial test kits (Meimian, Jiangsu, China). Optical density (OD) was selected at 450 nm, read by a microplate reader (Biotek, USA).

#### 2.5.9. Western Blotting for Protein Expression

The BCA kit was used to determine the concentrations of total protein extracted from the myocardium. After SDS-PAGE separation, equivalent quantities of cardiac tissue protein were transferred to NC membranes, which were then blocked in 5% skimmed milk powder for 2 h and incubated overnight at 4°C. Specific proteins were identified using antibodies against NLRP3, Caspase-1 p20, and GSDMD. The NC membranes were incubated overnight at 4°C, followed by 2 h at room temperature with the secondary antibodies. After washing 3 times with TBST, electrogenerated chemiluminescence (ECL) was used to develop and fix, and obtain the gel imager, and finally protein bands were obtained. The experiment was repeated 3 times. GAPDH was used as an internal control.

#### 2.5.10. RT-PCR for mRNA Expression

Total RNA was extracted from the left ventricular myocardium at 70 mg using the Trizol reagent and then reverse-transcribed to cDNA. RNA purity and quantification were determined based on its absorbance at 260 and 280 nm on a UV spectrophotometer. The cDNA was then analyzed using LightCycler® 96 PCR instrument (Roche, Switzerland). mRNA expression levels of NLRP3, Caspase-1, and GSDMD were analyzed using 2^−ΔΔCq^ methods, which were normalized to *β*-actin. The primer sequences used are shown in Table 1.

### 2.6. Statistical Analysis

All data results were expressed as mean ± SD and statistically analyzed by SPSS 23.0 software. One-way ANOVA and LSD tests were used to analyze the significance of the differences between groups. *P* < 0.05 was considered as significant difference.

## 3. Results

### 3.1. UPLC-PDA Qualitative Analysis

**Figure 1:**
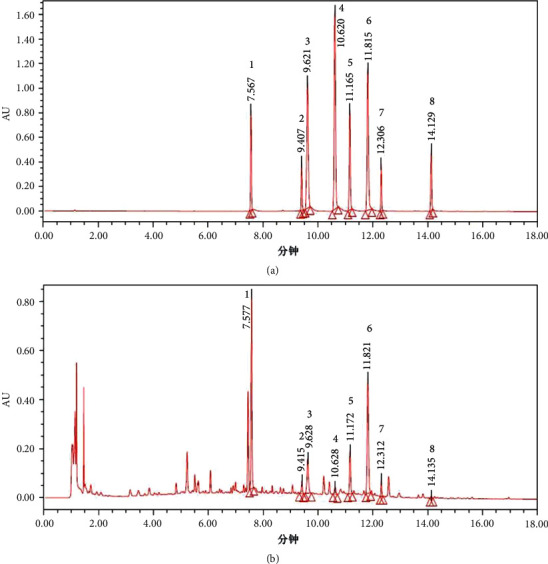
UPLC-PDA analysis of LGZGD. The chromatograms of standard compounds (a) and LGZGD (b). Liquiritin (**1**), isoliquiritin (**2**), coumarin (**3**), cinnamic alcohol (**4**), cinnamic acid (**5**), cinnamaldehyde (**6**), glycyrrhizic acid (**7**), and atractylenolide III (**8**).

**Figure 2:**
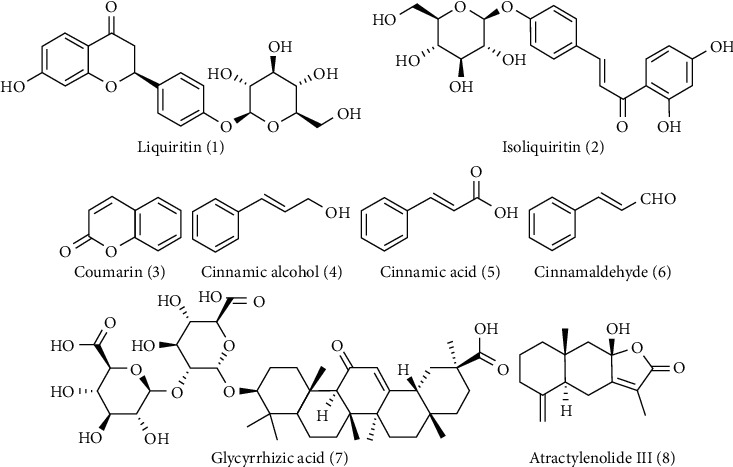
The characteristic chemical structures of LGZGD.

UPLC chromatogram of mixed standard compounds and LGZGD is shown in Figure 1. Eight authentic reference substances have been detected in LGZGD within 18 min. They are liquiritin (**1**), isoliquiritin (**2**), coumarin (**3**), cinnamic alcohol (**4**), cinnamic acid (**5**), cinnamaldehyde (**6**), glycyrrhizic acid (**7**), and atractylenolide III (**8**) (Figure 2).

### 3.2. Molecular Docking Results

#### 3.2.1. Docking of Main Components of LGZGD with NLRP3

**Figure 3:**
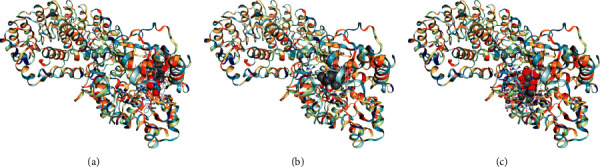
Molecular docking diagram of top 3 components of LGZGD and NLRP3: (a) liquiritin; (b) atractylenolide III; (c) isoliquiritin.

The main components of LGZGD had a good binding activity with NLRP3. Among them, the top three compounds were liquiritin, atractylenolide III, and isoliquiritin, which were close to the Vina score of the MCC950. The results suggested that LGZGD may act on NLRP3 (Table 2 and Figure 3).

#### 3.2.2. Docking of Main Components of LGZGD with Caspase-1

**Figure 4:**
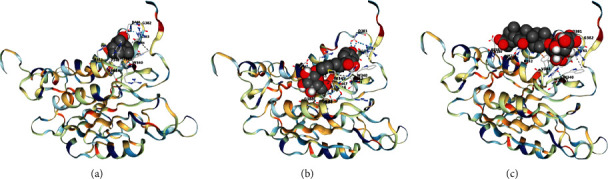
Molecular docking diagram of top 3 components of LGZGD and Caspase-1: (a) atractylenolide III; (b) isoliquiritin; (c) glycyrrhizic acid.

The main components of LGZGD had a good binding activity with Caspase-1. Among them, the top three compounds were atractylenolide III, isoliquiritin, and glycyrrhizic acid, and the Vina scores of atractylenolide III and isoliquiritin were better than that of ML132. These results indicate that LGZGD may act on Caspase-1, and atractylenolide III and isoliquiritin have strong potential activities and are worthy of further development (Table 3 and Figure 4).

#### 3.2.3. Docking of Main Components of LGZGD with GSDMD

**Figure 5:**
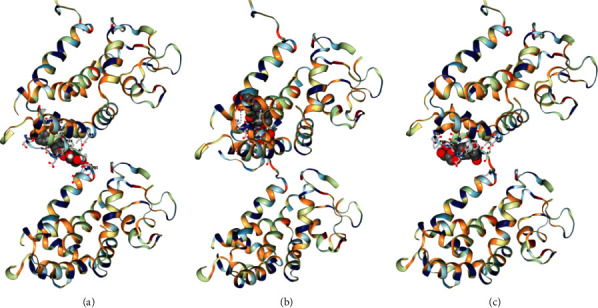
Molecular docking diagram of top 3 components of LGZGD and GSDMD: (a) isoliquiritin; (b) glycyrrhizic acid; (c) liquiritin.

The main components of LGZGD had a good binding activity with GSDMD. Among them, the top three compounds were isoliquiritin, glycyrrhizic acid, and liquiritin, which were close to the Vina score of the LDC7559. The results suggested that LGZGD may act on GSDMD (Table 4 and Figure 5). Molecular docking only predicted that LGZGD might act on NLRP3/Caspase-1/GSDMD signaling pathways, which was then verified by *in vivo* experiment.

### 3.3. In Vivo Experimental Results

#### 3.3.1. LGZGD Improves Cardiac Function after AMI

**Figure 6:**
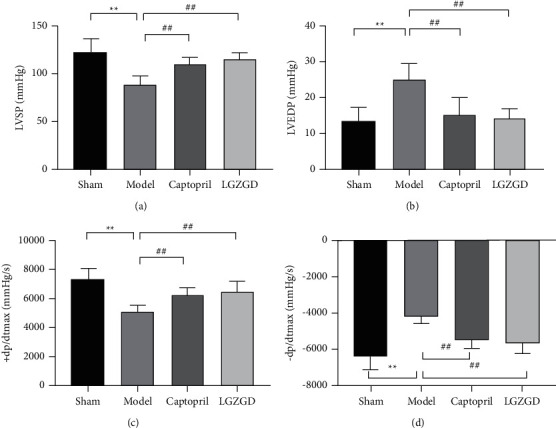
LGZGD improves cardiac function on hemodynamic indexes: (a) LVSP; (b) LVEDP; (c) +dP/dtmax; (d) −dP/dtmax. The values are expressed as mean ± SD (*n* = 10). ^*∗∗*^*P* < 0.01 vs. sham group, ^##^*P* < 0.01 vs. model group.

Compared with the sham group, LVSP, +dP/dtmax, and −dP/dtmax decreased significantly (*P* < 0.01), and LVEDP increased significantly in the model group (*P* < 0.01). LGZGD group and captopril group reversed the hemodynamic indexes (*P* < 0.01) (Figure 6). LGZGD could significantly improve cardiac function with AMI.

#### 3.3.2. LGZGD Improves the Morphological Changes via H&E Staining

**Figure 7:**
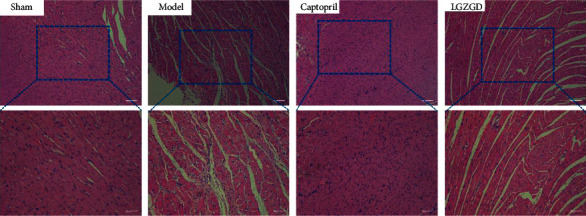
LGZGD improves the morphological changes via H&E staining (×100 and ×200).

As shown in Figure 7, the sham group's myocardial tissue was stained well, the morphology was normal, the texture was clear, the myocardial cells were organized in an ordered arrangement, and few cardiac fibroblasts were observed. The myocardium of the model group showed pathological changes with uneven staining and disordered arrangement, the proliferation of fibroblasts and infiltration of inflammatory cells. LGZGD and captopril could reduce the fibroblasts and alleviate the inflammatory cell infiltration. The results indicated that LGZGD could alleviate cardiac pathological changes and reduce the inflammatory response in AMI rats.

#### 3.3.3. LGZGD Alleviates Myocardial Fibrosis and CVF via Masson Staining

**Figure 8:**
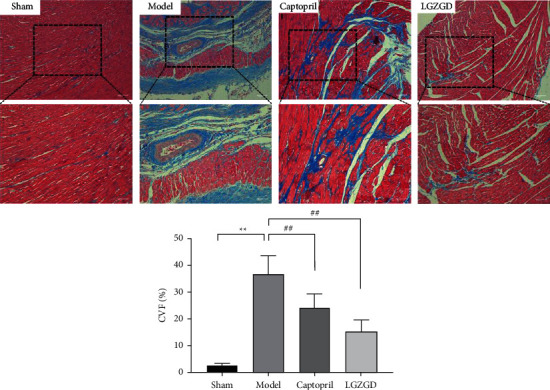
LGZGD alleviates myocardial fibrosis and CVF via Masson staining (×100 and ×200). Values are expressed as mean ± SD (*n* = 10). ^*∗∗*^*P* < 0.01 vs. sham group, ^##^*P* < 0.01 vs. model group.

The myocardial fibers were stained red, whereas the collagen fibers were stained blue using the Masson method. CVF can be used to observe the morphologic changes in the left ventricular myocardium. The myocardium was arranged neatly, with a few collagen fibers, and the distribution was relatively even in the sham group. In the model group, the arrangement of the myocardium was chaotic, the collagen was significantly increased, the distribution was disordered, and CVF was significantly increased (*P* < 0.01). The collagen of myocardial tissue was significantly reduced, the myocardial fibrosis was significantly improved, and CVF was significantly decreased in the LGZGD group and captopril group (*P* < 0.01) (Figure 8). LGZGD could reduce the excessive deposition of myocardial interstitial collagen.

#### 3.3.4. LGZGD Reduces Cardiomyocyte Apoptosis via TUNEL Staining

**Figure 9:**
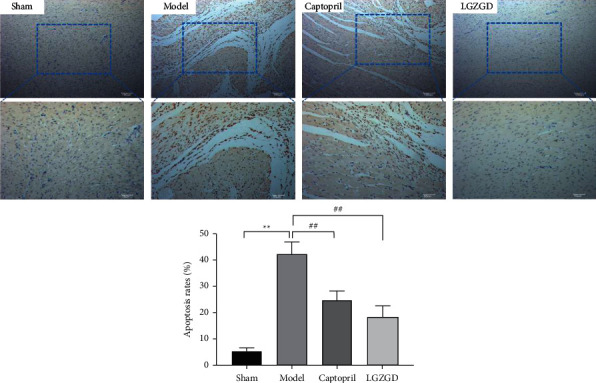
LGZGD reduces cardiomyocyte apoptosis via TUNEL staining. Light blue: the nucleus of normal myocardium; pale brown: the nucleus of apoptosis. The values are expressed as mean ± SD (*n* = 10). ^*∗∗*^*P* < 0.01 vs. sham group, ^##^*P* < 0.01 vs. model group.

Apoptosis rate was detected by TUNEL staining. The myocardial nucleus was pale brown after apoptosis (Figure 9). The myocardial nucleus in the model group was light brown, indicating that cardiomyocytes had apoptosis, and the apoptosis rate was significantly higher than those of the sham group (*P* < 0.01). The pale brown nuclei and apoptosis rate were considerably lower in the LGZGD and captopril group than in the model group (*P* < 0.01). The results indicated that LGZGD can significantly inhibit cardiomyocyte apoptosis in AMI rats.

#### 3.3.5. LGZGD Decreases NLRP3, Caspase-1, and GSDMD Levels via Immunohistochemical Analyses

**Figure 10:**
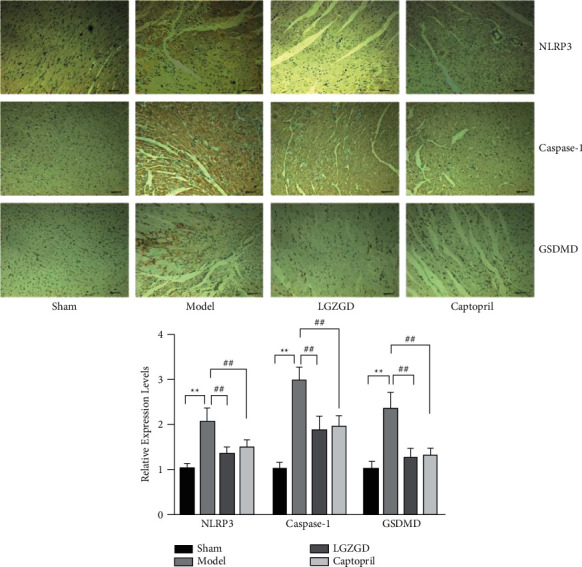
LGZGD decreases NLRP3, Caspase-1, and GSDMD levels via immunohistochemical analyses. The values are expressed as mean ± SD (*n* = 5). ^*∗∗*^*P* < 0.01 vs. sham group, ^##^*P* < 0.01 vs. model group.

NLRP3, Caspase-1, and GSDMD were brown and were diffused in the myocardial tissues via immunohistochemistry staining. The levels of NLRP3, Caspase-1, and GSDMD were significantly increased in the model group (*P* < 0.01). Compared with the model group, LGZGD and captopril groups had significantly decreased the expressions of NLRP3, Caspase-1, and GSDMD (*P* < 0.01). These data demonstrated that LGZGD significantly decreases the elevated NLRP3, Caspase-1, and GSDMD expression (Figure 10).

#### 3.3.6. LGZGD Decreases IL-1*β* Level in AMI-Induced Myocardial Injury

**Figure 11:**
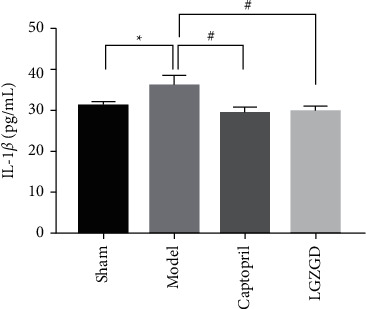
LGZGD decreases IL-1*β* level in AMI-induced myocardial injury. The values are expressed as mean ± SD (*n* = 10). ^*∗∗*^*P* < 0.05 vs. sham group, ^##^*P* < 0.05 vs. model group.

As shown in Figure 11, IL-1*β* level in rat serum increased significantly in the model group compared with the sham group (*P* < 0.05). IL-1*β* level in rat serum was decreased significantly in the LGZGD and captopril groups (*P* < 0.05).

#### 3.3.7. LGZGD Regulates Protein Expressions of NLRP3, Caspase-1, and GSDMD in AMI Rats

**Figure 12:**
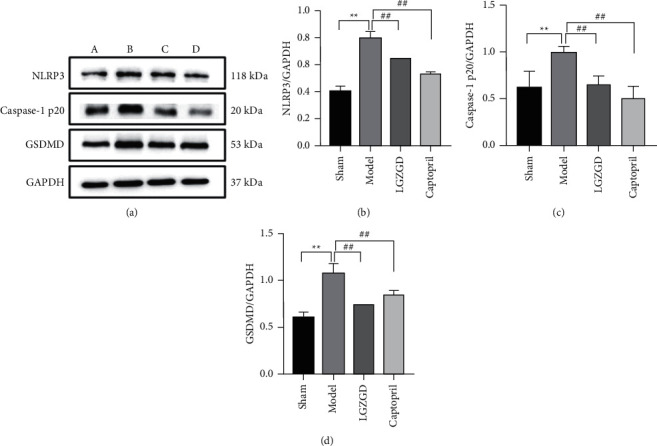
LGZGD regulates protein expressions of NLRP3, Caspase-1, and GSDMD in AMI rats: (a) NLRP3, Caspase-1, and GSDMD pictures of Western spots; (b) NLRP3; (c) Caspase-1 p20; (d) GSDMD. (A) sham group; (B) model group; (C) LGZGD group; (D) captopril group. The values were expressed as the mean ± SD (*n* = 3). ^*∗∗*^*P* < 0.01 vs. sham group, ^##^*P* < 0.01 vs. model group.

In comparison to the sham group, the model group dramatically enhanced the expression of NLRP3, Caspase-1, and GSDMD proteins (*P* < 0.01). LGZGD and captopril were shown to be effective in reversing protein overexpression (*P* < 0.01, *P* < 0.05) (Figure 12). LGZGD could inhibit the expression of related proteins in the NLRP3/Caspase-1/GSDMD signaling pathway.

#### 3.3.8. LGZGD Inhibits the Expression of NLRP3, Caspase-1, and GSDMD mRNA in AMI Rats

**Figure 13:**
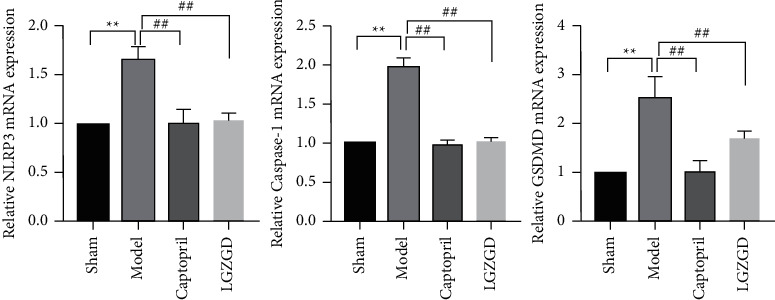
LGZGD inhibits the expression of NLRP3, Caspase-1, and GSDMD mRNA in AMI rats. The values were expressed as the mean ± SD (*n* = 3). ^*∗∗*^*P* < 0.01 vs. sham group, ^##^*P* < 0.01 vs. model group.

The RT-qPCR analysis revealed the expression of genes. NLRP3, Caspase-1, and GSDMD mRNA expression levels were markedly higher in the model group than in the sham group (*P* < 0.01). LGZGD and captopril have the ability to reverse mRNA expressions (*P* < 0.01) (Figure 13). LGZGD treatment can significantly inhibit NLRP3/Caspase-1/GSDMD signaling pathway-related gene expression in the AMI model rats, which is consistent with the effect on protein expression.

## 4. Discussion

AMI induces a strong inflammatory response that activates NF-*κ*B. Activated NF-*κ*B translocation to the nucleus regulates the transcription of inflammatory and immune-related genes, induces the expression of cytokines, and then interferes with the differentiation, proliferation, and apoptosis of cardiomyocytes, which induces the inflammatory cascade reaction and accelerates the pathological process of VR [31, 32]. Additionally, activation of NF-*κ*B elevated the expression of NLRP3 in cardiomyocytes. NLRP3 is one of the inflammasomes, consisting of NLRP3, ASC (apoptosis-associated speck-like protein containing a caspase recruitment domain), and Procaspase-1 [33, 34]. In response to the pyroptosis signal, the pattern recognition receptors in the cell bind to the associated pattern recognition molecules, and NLRP3 remains inactive in the cytoplasm. The PYD domain of NLRP3 interacts with the ASC, and the ASC's CARD domain interacts with Procaspase-1, resulting in the formation of the complete NLRP3 inflammasome, NLRP3-ACS-Procaspase-1. Then, it stimulates downstream Caspase-1 targets, resulting in Caspase-1 cleavage and activation, induction of maturation, and secretion of IL-1*β* [35, 36]. NLRP3 activation has a critical role in the onset and progression of CVDs. Following AMI initiation, ischemia damage, cell death, and related cell debris activate the inflammasome, resulting in the inflammatory response [37, 38]. NLRP3 inhibitor can significantly reduce myocardial infarct area, maintain cardiac function, and inhibit the overactivation of NLRP3 inflammasome of AMI animal models [39].

Recent research indicates that activation of the NLRP3 inflammasome has a role in basic structural alterations in VR after AMI [40]. Cardiac pressure overload causes Ca^2+^ influx and triggers the activation of NLRP3 inflammasome in cardiomyocytes, which results in the recruitment of cardiac macrophages, the formation of fibrosis, and the development of myocardial dysfunction [41]. After 2 weeks of treatment with NLRP3 inflammasome inhibitor MCC950 in MI/R mice, myocardial fibrosis and NLRP3-induced pyroptosis were significantly reduced [42]. P2X7 inhibition (using silencing RNA) could prevent the formation of the NLRP3 inflammasome, limit infarct size and cardiac enlargement in AMI, and alleviate VR on the 7th day, which was characterized by slight ventricular dilatation and dysfunction [43]. These results suggest that NLRP3-inflammasome-mediated pyroptosis may be an important target for drug therapy for VR after AMI [44]. To alleviate AMI following VR, it is necessary to inhibit the activity of the NLRP3 inflammasome and reduce the overexpression of inflammatory factors. Therefore, a new approach to prevent and treat VR after AMI can be initiated by inhibiting NLRP3 activation, and classical TCM prescriptions with definite curative effects can be sought.

In this study, eight main components of LGZGD were determined by UPLC, namely, liquiritin, isoliquiritin, coumarin, cinnamic alcohol, cinnamic acid, cinnamaldehyde, glycyrrhizic acid, and atractylenolide III, which are consistent with the main chemical components of LGZGD previously reported [45]. CB-dock is a simple blind docking Web server that predicts the binding situation and places of small molecules in a given target. The outcomes of interactive 3D visualization between molecules and targets, as well as the binding of chemicals to amino acid residues, may be visibly given. The results of molecular docking showed that the main components of LGZGD may act on the major targets of the NLRP3/Caspase-1/GSDMD signaling pathway. Among the main components of LGZGD, the compound that could bind to NLRP3, Caspase-1, and GSDMD was isoliquiritin. In addition, liquiritin could bind to NLRP3 and GSDMD. Isoliquiritin is likely to act as a lead compound in the inhibitory of NLRP3/Caspase-1/GSDMD signaling pathway on AMI. Isoliquiritin and liquiritin are typical flavonoids, and flavonoids are beneficial to cardiovascular diseases, an important part of “functional food,” which, by enhancing cardiac functioning, can decrease cardiomyocyte apoptosis and fibrotic marker generation and prevent cardiac hypertrophy [46]. The results of experiments showed that isoliquiritin has a good inhibitory effect on NF-*κ*B, NLRP3, cleaved Caspase-1, and IL-1*β* [47]. In addition, atractylenolide III could decrease the expressions of cleaved Caspase-1 and NLRP3, indicating that atractylenolide III reduced NLRP3 inflammasome activation in ovalbumin-induced asthmatic mice [48]. Glycyrrhizic acid could decrease the increased levels of NLRP3, ASC, and Caspase-1 induced by HMGB1 and inhibit IL-1*β* production [49]. Cinnamic acid can significantly inhibit NLRP3, NF-*κ*B, and ASK1/MAPK signaling pathways, thus alleviating oxidative stress injury of pancreas [50]. Cinnamaldehyde can reduce the expressions of TLR4, NF-*κ*B, NLRP3, Caspase-1, and IL-1*β* in the prefrontal cortex and hippocampus, which inhibited the NF-*κ*B pathway and NLRP3 inflammasome overactivation [51]. Since molecular docking shows that the main chemical components of LGZGD have good binding ability with the proteins on the NLRP3/Caspase-1/GSDMD signal pathway, and the references also reported that some chemical components could act on NLRP3, it is particularly important to further study the correlation between LGZGD and the NLRP3/Caspase-1/GSDMD signal pathway *in vivo*.

The results of our pharmacodynamic experiments showed that LGZGD could significantly improve cardiac function and cardiac pathological changes, reduce the excessive deposition of myocardial interstitial collagen, and inhibit cardiomyocyte apoptosis. LGZGD could significantly reduce IL-1*β* release after activation of the NLRP3/Caspase-1/GSDMD signaling pathway. Additional investigations demonstrated that LGZGD may drastically reduce the levels of mRNA and protein expression of the targets in the AMI-activated NLRP3/Caspase-1/GSDMD signaling pathway. LGZGD may reduce NLRP3 inflammasome activation, therefore inhibiting the NLRP3/Caspase-1/GSDMD signaling pathway.

## 5. Conclusion

In summary, UPLC combined with molecular docking technology preliminarily elucidates the main components of LGZGD and its mechanism of inhibiting the NLRP3/Caspase-1/GSDMD signal pathway. In this study, modern molecular docking technology was used to predict the mechanism of TCM in preventing and treating VR after AMI, providing a new method for the study of TCM mechanisms. *In vivo* experimental results showed that LGZGD could significantly improve cardiac function, alleviate myocardial fibrosis, reduce the level of inflammatory factors, and inhibit the activation of NLRP3/Caspase-1/GSDMD signaling pathway. The highlight of this study is that UPLC and molecular docking technology were used to predict the targets of LGZGD, and *in vivo* experiments were used to further verify the correlation between LGZGD and the NLRP3/Caspase-1/GSDMD signal pathway, which is a combination of theory and practice. However, we only investigated a portion of the mechanism of LGZGD in the prevention and treatment of VR after AMI, and we did not employ cardiomyocytes, gene silencing, or overexpression to carry out in-depth investigation. In the future study, cardiomyocytes will be employed to conduct in-depth research on the specific regulatory target and mechanism of LGZGD, to further explain the mechanism of LGZGD in the prevention and treatment of VR after AMI.

## Figures and Tables

**Table 1:** Primers used in qRT-PCR.

Primers		Sequence (5′ ⟶ 3′)
NLRP3	Forward Reverse	5′-GAGCTGGACCTCAGTGACAATGC-3′ 5′-AGAACCAATGCGAGATCCTGACAAC-3′
Caspase-1	Forward Reverse	5′-GCACAAGACTTCTGACAGTACCTTCC-3′ 5′-GCTTGGGCACTTCAATGTGTTCATC-3′
GSDMD	Forward Reverse	5′-CAGCAGGCAGCATCCTTGAGTG-3′ 5′-CCTCCAGAGCCTTAGTAGCCAGTAG-3′
*β*-Actin	Forward Reverse	5′-CCCGCGAGTACAACCTTCTTG-3′ 5′-GTCATCCATGGCGAACTGGTG-3′

**Table 2:** Docking results of main components of LGZGD with NLRP3.

Chemicals	Vina score	Cavity score	Center (*x*, *y*, *z*)	Size (*x*, *y*, *z*)
MCC950	−10.4	12730	88, 94, 81	35, 34, 35
**Liquiritin**	**−9.4**	**12730**	**88**, **94**, **81**	**35**, **34**, **35**
**Atractylenolide III**	**−8.9**	**12730**	**88**, **94**, **81**	**35**, **34**, **35**
**Isoliquiritin**	**−8.2**	**12730**	**88**, **94**, **81**	**35**, **34**, **35**
Glycyrrhizic acid	−6.5	12730	88, 94, 81	35, 29, 35
Coumarin	−6.5	12730	88, 94, 81	35, 34, 35
Cinnamic acid	−5.9	12730	88, 94, 81	35, 34, 35
Cinnamic alcohol	−5.7	12730	88, 94, 81	35, 34, 35
Cinnamaldehyde	−5.7	12730	88, 94, 81	35, 34, 35

Bold represents the top three compounds' Vina scores.

**Table 3:** Docking results of main components of LGZGD with Caspase-1.

Chemicals	Vina score	Cavity score	Center (*x*, *y*, *z*)	Size (*x*, *y*, *z*)
ML132	−6.8	685	41, 65, 6	24, 24, 24
**Atractylenolide III**	**−8.2**	**685**	**41**, **65**, **6**	**19**, **19**, **19**
**Isoliquiritin**	**−7.9**	**685**	**41**, **65**, **6**	**27**, **27**, **27**
**Glycyrrhizic acid**	**−7.3**	**685**	**41**, **65**, **6**	**29**, **29**, **29**
Liquiritin	−7.1	685	41, 65, 6	24, 24, 24
Coumarin	−6.0	685	41, 65, 6	17, 24, 24
Cinnamic acid	−5.7	685	41, 65, 6	18, 24, 24
Cinnamaldehyde	−5.5	685	41, 65, 6	18, 24, 24
Cinnamic alcohol	−5.4	685	41, 65, 6	18, 24, 24

Bold represents the top three compounds' Vina scores.

**Table 4:** Docking results of main components of LGZGD with GSDMD.

Chemicals	Vina score	Cavity score	Center (*x*, *y*, *z*)	Size (*x*, *y*, *z*)
LDC7559	−7.5	868	30, −83, 41	24, 24, 24
**Isoliquiritin**	**−7.0**	**868**	**30**, **−83**, **41**	**27**, **27**, **27**
**Glycyrrhizic acid**	**−6.7**	**868**	**30**, **−83**, **41**	**29**, **29**, **29**
**Liquiritin**	**−5.7**	**868**	**30**, **−83**, **41**	**24**, **24**, **24**
Atractylenolide III	−5.4	868	30, −83, 41	19, 19, 25
Coumarin	−5.3	868	30, −83, 41	17, 17, 25
Cinnamic acid	−4.8	868	30, −83, 41	18, 18, 25
Cinnamic alcohol	−4.6	868	30, −83, 41	18, 18, 25
Cinnamaldehyde	−4.4	868	30, −83, 41	18, 18, 25

Bold represents the top three compounds' Vina scores.

## Data Availability

The data used to support the findings of this study are available from the corresponding author upon request.
